# Supplementary material for: Interstitial Photodynamic Therapy of Glioblastomas: A Long-Term Follow-up Analysis of Survival and Volumetric MRI Data
Source: Cancers (Basel). 2023 May 4;15(9):2603. doi: 10.3390/cancers15092603 (PMC10177153; doi:10.3390/cancers15092603)
Supplement: Supplementary file 1 [file cancers-15-02603-s001.zip › cancers-2233155-supplementary.pdf]

Supplementary figures and tables

Interstitial photodynamic therapy of glioblastoma: An MRI-based follow-up analysis

Foglar, M.; Aumiller, M.; Bochmann, K.; Buchner, A.; El Fahim, M.; Quach, S.; Sroka, R.; Stepp, H.; Thon, N.; Forbrig, R. and Rühm, A.

Supplementary Figure S1

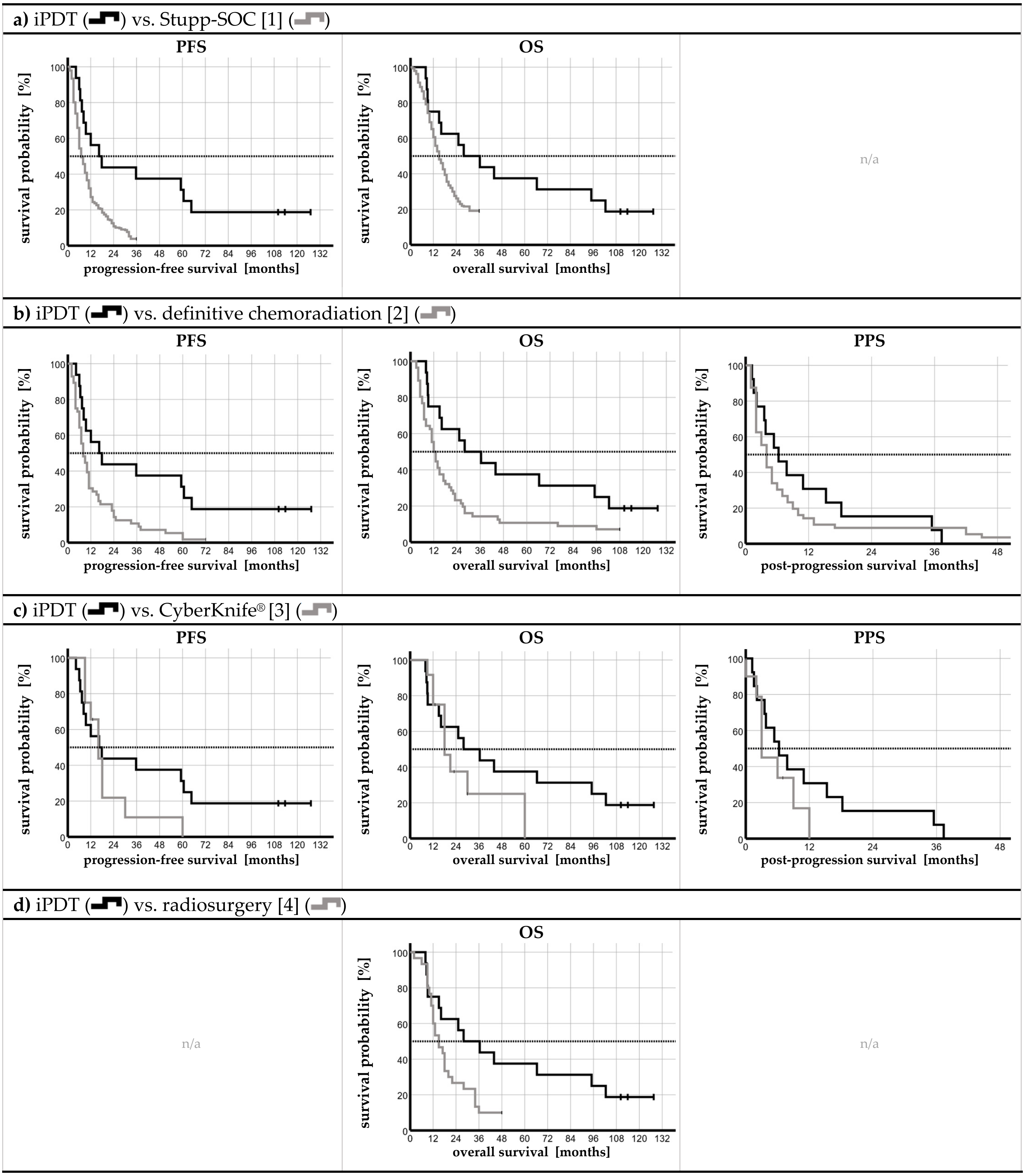

PFS = progression-free survival; **OS** = overall survival; **PPS** = post-progression survival

Supplementary Figure S1: Kaplan-Meier plots for comparative outcome evaluation of the iPDT-treated patient cohort and patient cohorts receiving other types of treatment. **a)**, **b)** and **d)** were reconstructed from published Kaplan-Meier plots, for **c)** published data were used directly. All graphs marked with "n/a" did not qualify for analysis due to missing data in the original publications.

Supplementary Table S1

IPDT no. (⟳PFS, †OS)	tumor pre iPDT [mm³]	contrast enhancement pre iPDT [mm³]	necrosis pre iPDT [mm³]	NTR pre iPDT [mm³/mm³]	contrast enhancement 1 day post iPDT [mm³]	iPDT remnant 1 day post iPDT [mm³]	iPDT remnant 3 months post iPDT [mm³]	iPDT remnant 3 months pre recurrence [mm³]	iPDT remnant at recurrence [mm³]	recurrent tumor at recurrence [mm³]
**IPDT 01** (⟳65, †102)	1678	1364	314	0.19	0	6407	n/a	n/a	953	208
**IPDT 02** (⟳59; †95)	1362	878	484	0.36	22	2062	370	305	157	261
**IPDT 03** (128)	5207	2721	2485	0.48	691	12426	n/a	n/a	n/a	n/a
**IPDT 04** (⟳8, †15)	8574	7708	865	0.10	347	43691	n/a	12254	6263	10780
**IPDT 05** (⟳12, †16)	2604	1592	1013	0.39	387	22270	3940	4036	5221	15683
**IPDT 06** (⟳4, †9)	1951	1834	118	0.06	82	6381	n/a	n/a	3157	2070
**IPDT 07** (110)	2269	2059	210	0.09	n/a	n/a	6599	n/a	n/a	n/a
**IPDT 08** (⟳61, †66)	4079	2819	1260	0.31	535	11440	3975	2852	2852	11
**IPDT 09** (114)	14263	9644	4619	0.32	0	66857	20027	n/a	n/a	n/a
**IPDT 10** (⟳16, †28)	6920	3827	3093	0.45	2695	6666	5721	5209	9131	17177
**IPDT 11** (⟳6, †9)	11023	5965	5058	0.46	805	18977	10087	n/a	7335	6165
**IPDT 12** (⟳7, †8)	13692	12200	1492	0.11	1581	15241	4742	n/a	n/a	n/a
**IPDT 13** (⟳10, †25)	7176	5735	1441	0.20	125	11552	1277	4975	4670	5621
**IPDT 14** (⟳36, †44)	15335	8929	6406	0.42	4720	22917	8689	5464	9607	6
**IPDT 15** (⟳7, †9)	3990	3239	750	0.19	569	8833	5448	3548	2994	13197
**IPDT 16** (⟳18, †36)	21825	12362	9463	0.43	4054	31773	14935	1625	2335	8557

PFS = ⟳ = progression-free survival; **OS** = † = overall survival; **n/a** = MR imaging not available; **0** = volume was not present; **NTR** = Necrosis-tumor ratio

Supplementary Table S1: Individual tumor-related volumes and volume ratios (values rounded to full mm³, suppl. for **Table 5**)

Supplementary Table S2

		in relation to	p-value	HR	95 % CI
**a)**	age at diagnosis [years]	OS	0.067	1.038	[0.997; 1.080]
**b)**	cycles of TMZ [n]	OS	0.146	0.859	[0.700; 1.055]
**c)**	NTR [mm³/mm³]	OS	0.540	0.279	[0.005; 16.533]
**d)**	PPS [months]	PFS	0.053	0.938	[0.879; 1.001]
**e)**	tumor volume [cm³]	OS	0.653	1.020	[0.935; 1.112]
**f)**	CE volume 24h post-iPDT [cm³]	OS	0.599	1.096	[0.779; 1.541]
**g)**	iPDT remnant 24h post-iPDT [cm³]	OS	0.401	0.986	[0.954; 1.019]
**h)**	iPDT remnant 3 months post-iPDT [cm³]	OS	0.341	0.946	[0.845; 1.060]
**i)**	iPDT remnant 3 months before recurrence [cm³]	OS	0.051	1.318	[0.998; 1.740]
**k)**	iPDT remnant at recurrence [cm³]	OS	0.156	1.144	[0.950; 1.377]
**l)**	presence of CE 3 months post-iPDT [yes; no]	OS	0.223	2.259	[0.610; 8.363]
**m)**	MGMT-status [yes/partially; no]	OS	0.042	0.280	[0.082; 0.956]

HR = hazard ratio; **a)** – **k)**: HR per step in unit listed; **l)** – **m)**: HR by category

Supplementary Table S2: Univariate Cox regression analysis for possibly survival-related factors displayed in Figures 4 to 6

Supplementary Table S3

		Stupp [1]		Chemoradiation [2]		CyberKnife® [3]		Stereotactic radiosurgery [4]	
		published values	calculated values	published values	calculated values	published values	calculated values	published values	calculated values
PFS [months]	median [95 % CI]	6.9 [5.8; 8.2]	7.0 [6.1; 7.9]	8.1 [n/a; n/a]	8.0 [5.6; 10.4]	16 [n/a; n/a]	16.0 [10.4; 21.6]	8.2 [4.6; 10.5]	n/a
OS [months]	median [95 % CI]	14.6 [13.2; 16.8]	15.0 [13.3; 16.7]	12.1 [n/a; n/a]	12.0 [9.6; 14.4]	18 [n/a; n/a]	18.0 [10.9; 25.1]	14.8 [10.9; 19.9]	15.0 [9.6; 20.4]
PPS [months]	median [95 % CI]	n/a	n/a	3.4 [n/a; n/a]	4.0 [3.0; 5.0]	n/a	3.0 [2.0; 4.0]	n/a	n/a

PFS = progression-free survival; **OS** = overall survival; **PPS** = post-progression survival

Supplementary Table S3: Survival times (median, [95 % CI]) for different treatments reported in literature, with comparison of published values and re-calculated values based on digitized Kaplan-Meier graphs (values rounded)

References

1. Stupp, R., et al., *Radiotherapy plus concomitant and adjuvant temozolomide for glioblastoma.* N Engl J Med, 2005. **352**(10): p. 987-96.
2. Thon, N., et al., *Outcome in unresectable glioblastoma: MGMT promoter methylation makes the difference.* J Neurol, 2017. **264**(2): p. 350-358.
3. Oermann, E., et al., *CyberKnife enhanced conventionally fractionated chemoradiation for high grade glioma in close proximity to critical structures.* J Hematol Oncol, 2010. **3**: p. 22.
4. Azoulay, M., et al., *A phase I/II trial of 5-fraction stereotactic radiosurgery with 5-mm margins with concurrent temozolomide in newly diagnosed glioblastoma: primary outcomes.* Neuro Oncol, 2020. **22**(8): p. 1182-1189.
